# Supplementary material for: Exploration of a European-centered strawberry diversity panel provides markers and candidate genes for the control of fruit quality traits
Source: Hortic Res. 2024 May 14;11(7):uhae137. doi: 10.1093/hr/uhae137 (PMC11233882; doi:10.1093/hr/uhae137)
Supplement: Web_Material_uhae137 [file web_material_uhae137.pdf]

Exploration of a European-centered strawberry diversity panel provides markers and candidate genes for the control of fruit quality traits

Alexandre Prohaska[1,2], Pol Rey-Serra[1], Johann Petit[1], Aurélie Petit[2], Justine Perrotte[2], Christophe Rothan[1*], Béatrice Denoyes[1*]

Supplementary File

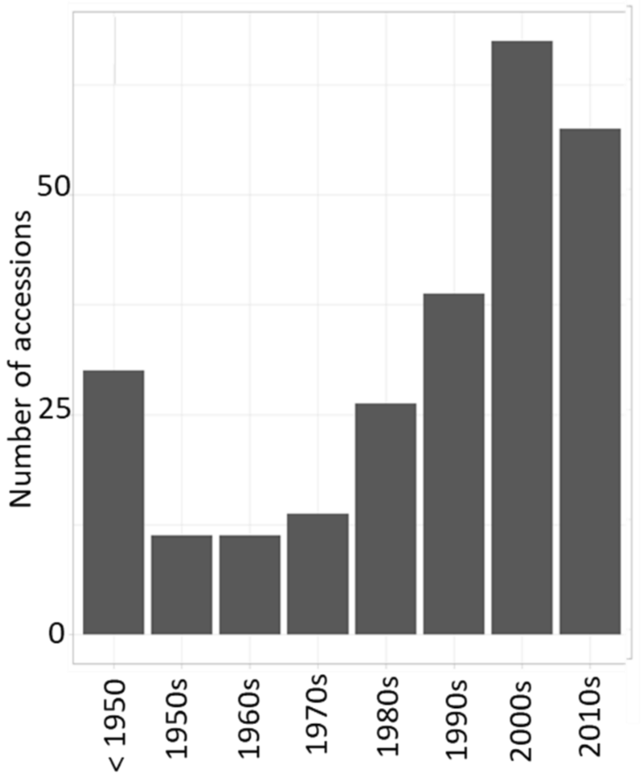

S**upplementary Figure S1**. Distribution of the year of release for the 223 accessions of the diversity panel.

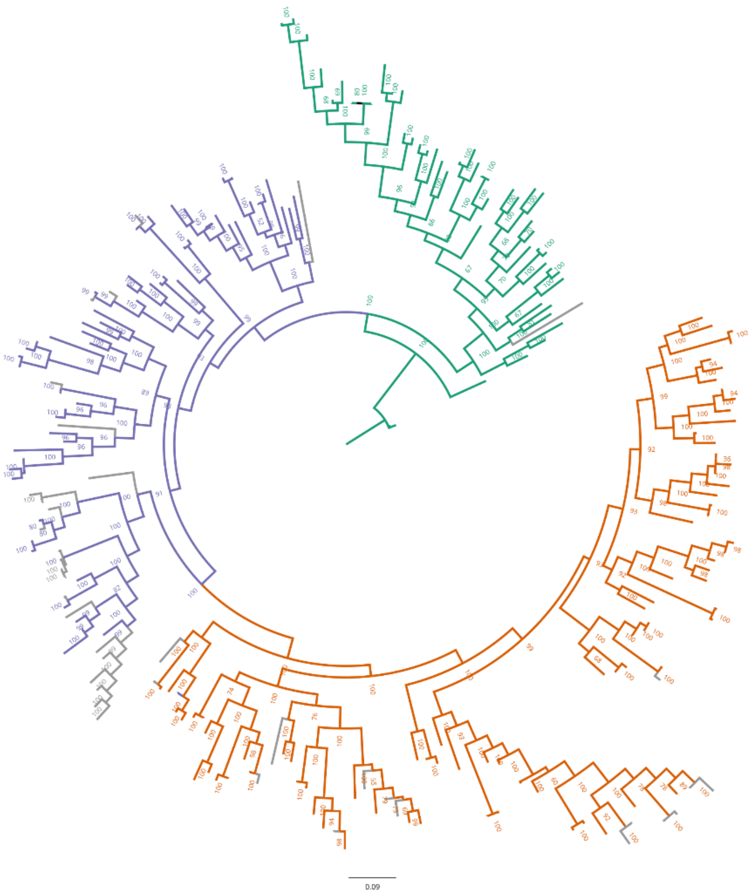

Supplementary Figure S2. Phylogenetic tree of the 223 accessions of the diversity panel using the TVMe+ASC+R3 model. The genetic group 1 is colored in green, group 2 in purple and group 3 in orange. Individuals clustered within the wrong group are labeled in grey. Groups 1, 2, 3: Heirloom & related, European mixed group and American & European mixed groups, respectively.

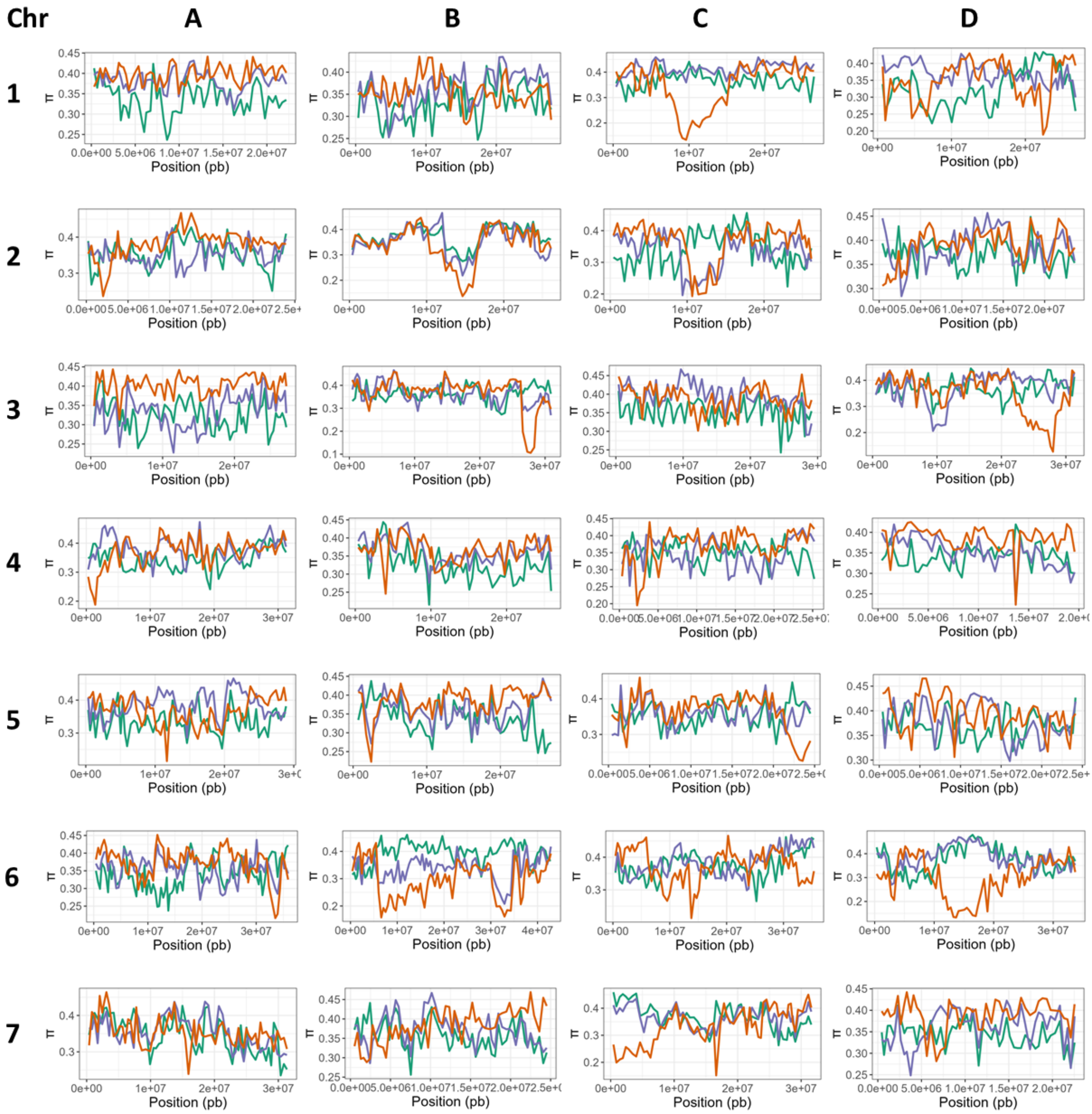

Supplementary Figure S3. π chromosome-wide estimates for each genetic group for 400kb windows across the octoploid genome. The genetic group 1 is colored in green, group 2 in purple and group 3 in orange. Groups 1, 2, 3: Heirloom & related, European mixed group and American & European mixed groups, respectively.

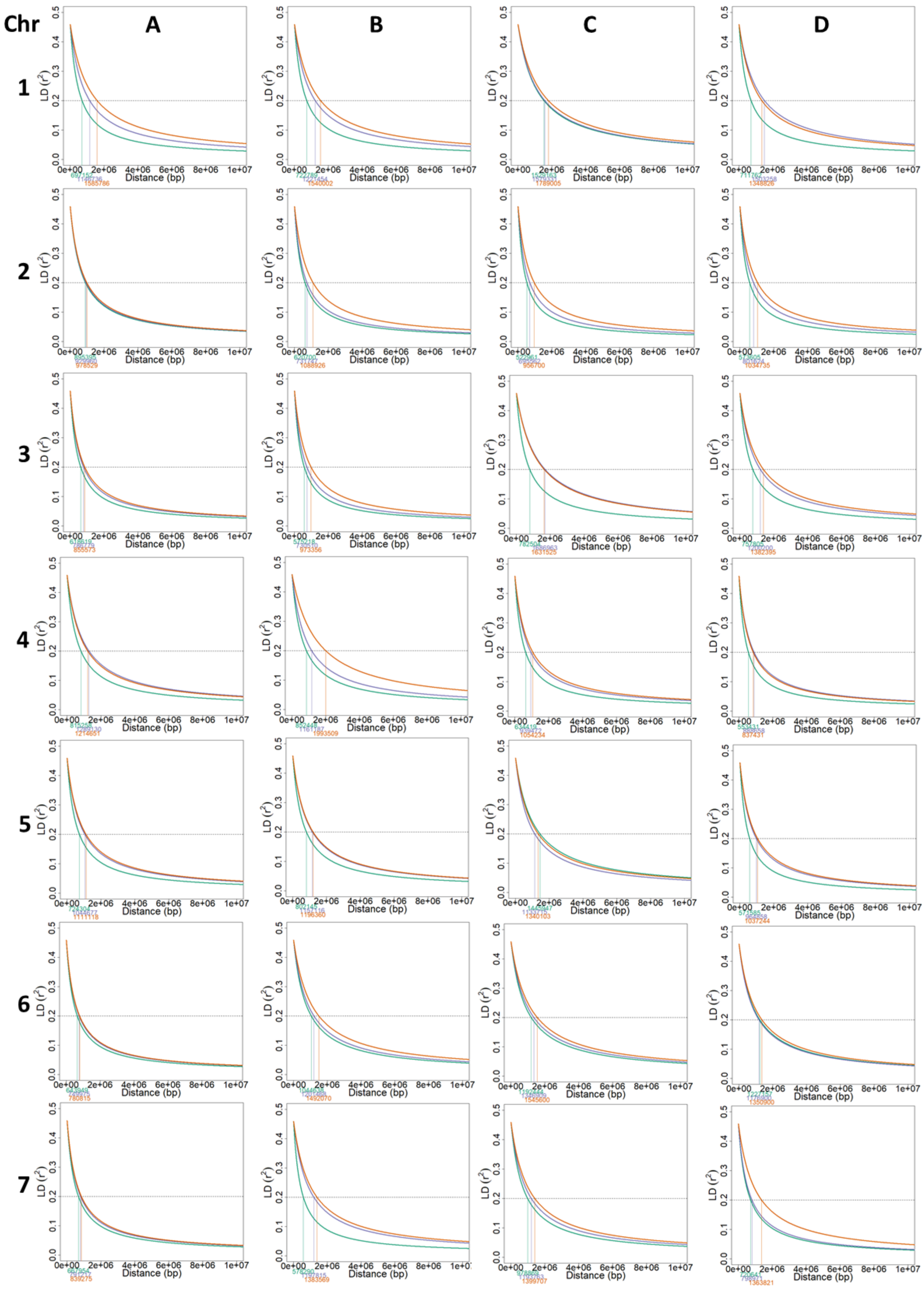

Supplementary Figure S4. Linkage disequilibrium (LD) decay along each chromosome of the octoploid genome. The genetic group 1 is colored in green, group 2 in purple and group 3 in orange. The dashed line represents the LD decay at r2 = 0.2. Groups 1, 2, 3: Heirloom & related, European mixed group and American & European mixed groups, respectively

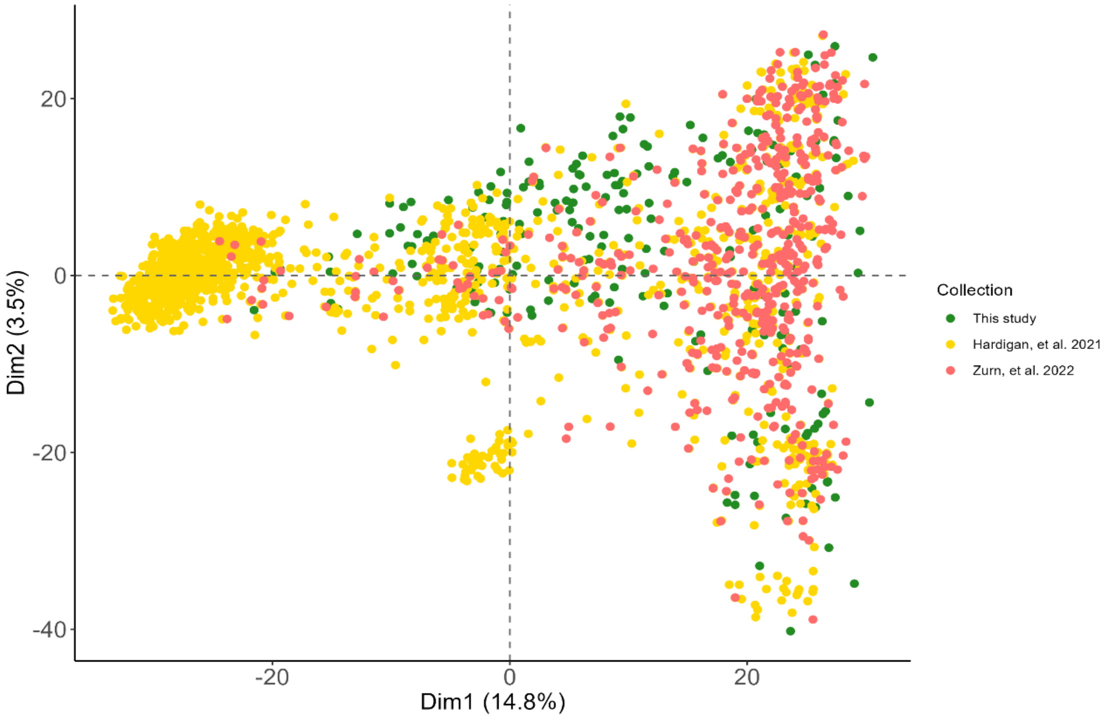

Supplementary Figure S5. Distribution of the Invenio panel (green dots) among published data. 1 569 genotypes (yellow dots) studied in Hardigan et al. (2020) and 539 genotypes studied in Zurn et al. (2022) (red dots) with 3 215 SNP markers.

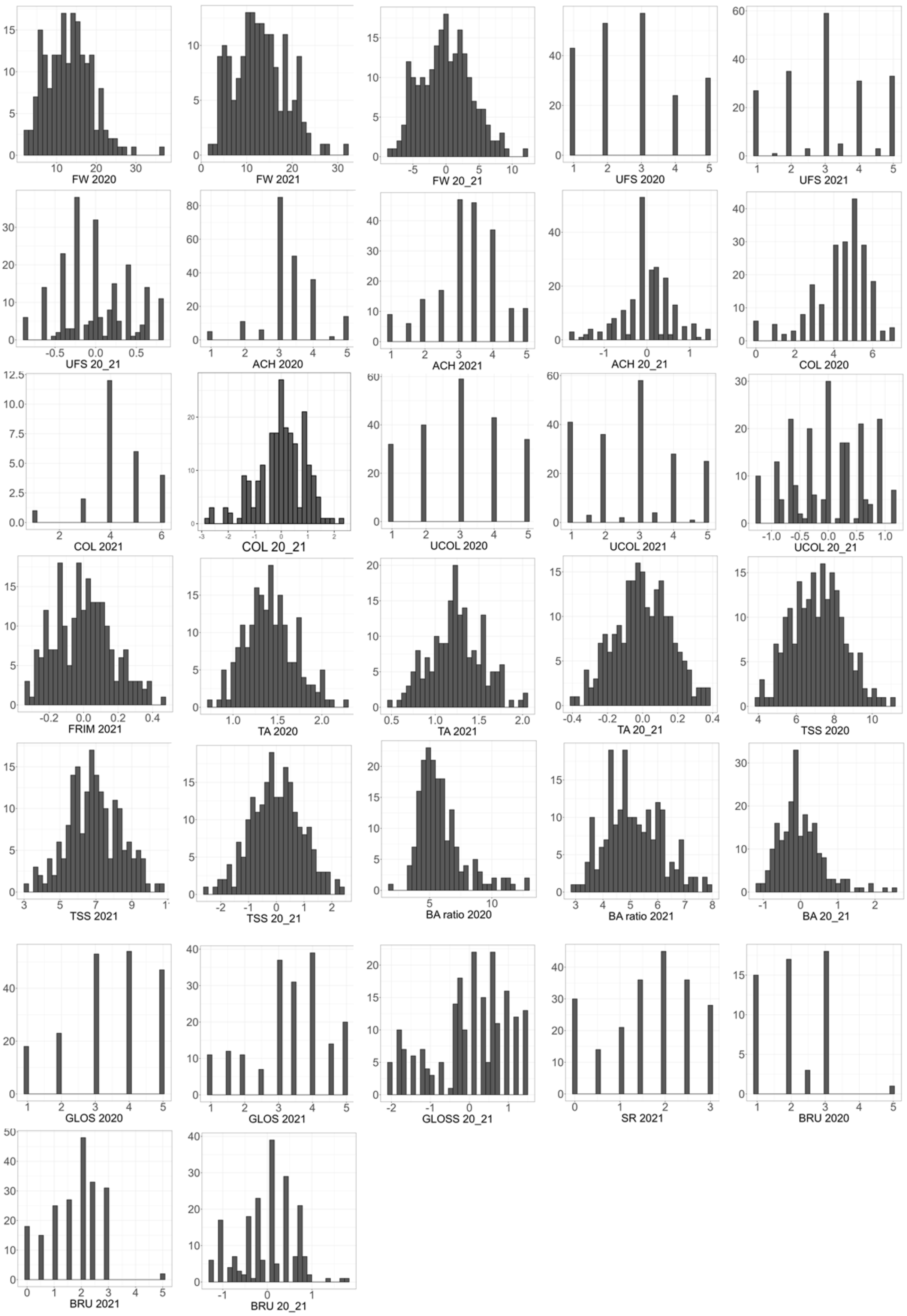

Supplementary Figure S6. Distribution of BLUP estimates for the 12 traits. FW, fruit weight; UFS, uniformity of fruit shape; COL, skin color; UCOL, uniformity of skin color; ACH, position and depth of achenes; FIRM, firmness; TA, titratable acidity; TSS, total soluble solids; BA, Brix/TA ratio; GLOS, glossiness; SR, skin resistance; BRU, bruisedness.

A

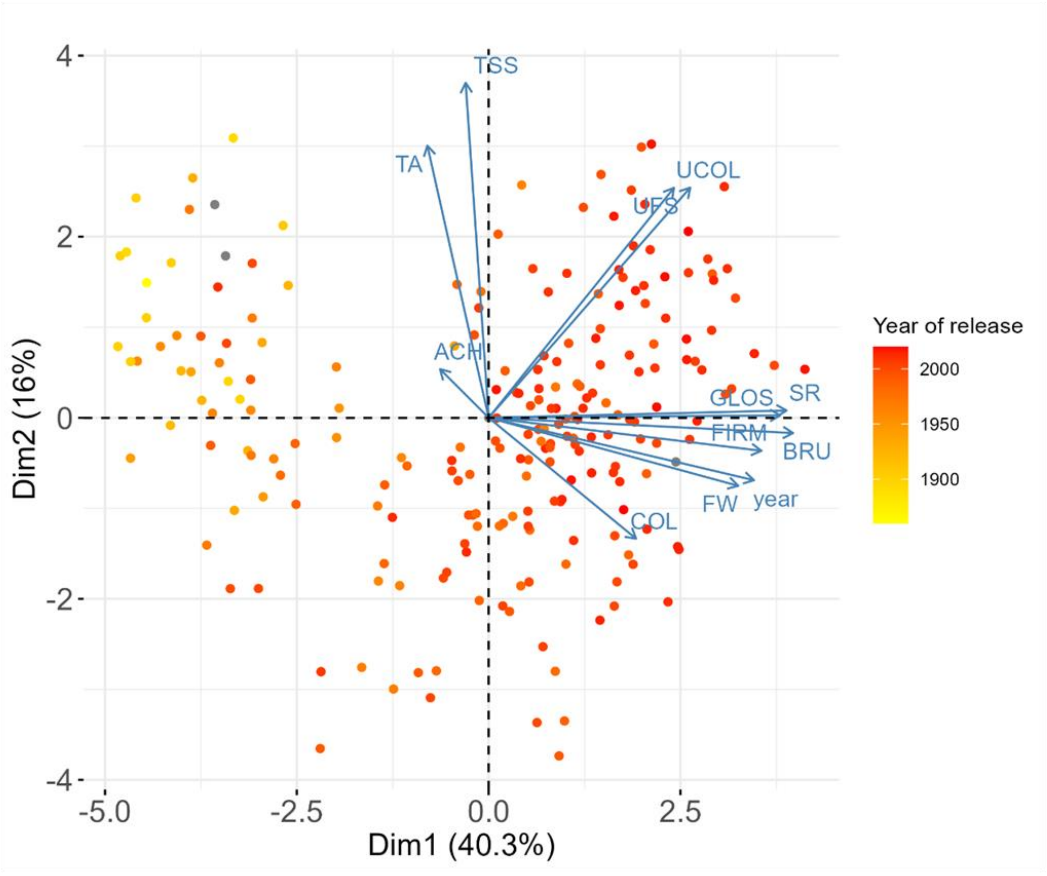

B

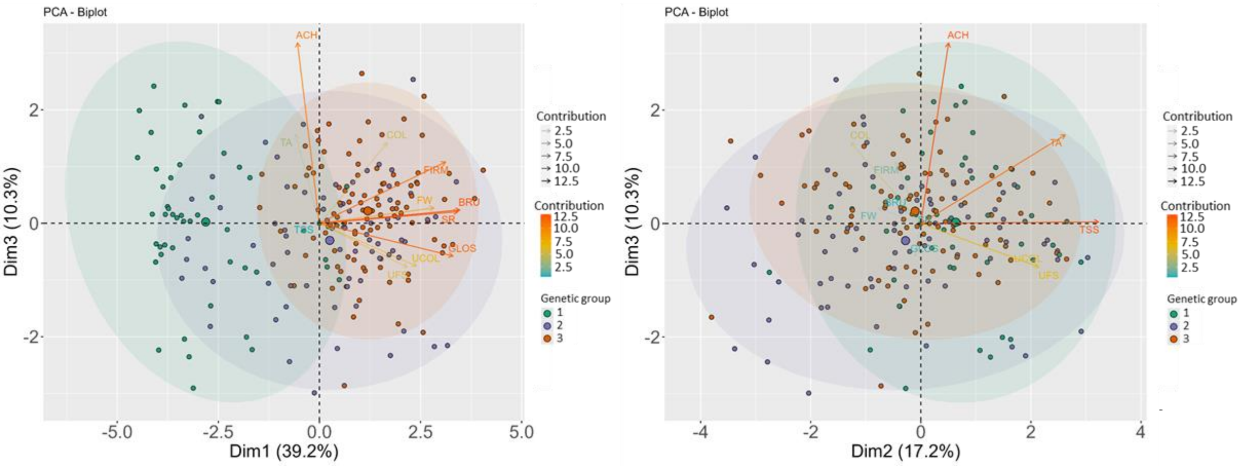

Supplementary Figure S7. Principal Component Analysis of the 2-year BLUP values for 11 traits. (A,B) Each accession (dot) is colored by its year of release (A) or its genetic group (B).FW, fruit weight; UFS, uniformity of fruit shape; COL, skin color; UCOL, uniformity of skin color; ACH, position and depth of achenes; FIRM, firmness; TA, titratable acidity; TSS, total soluble solids; GLOS, glossiness; SR, skin resistance; BRU, bruisedness.

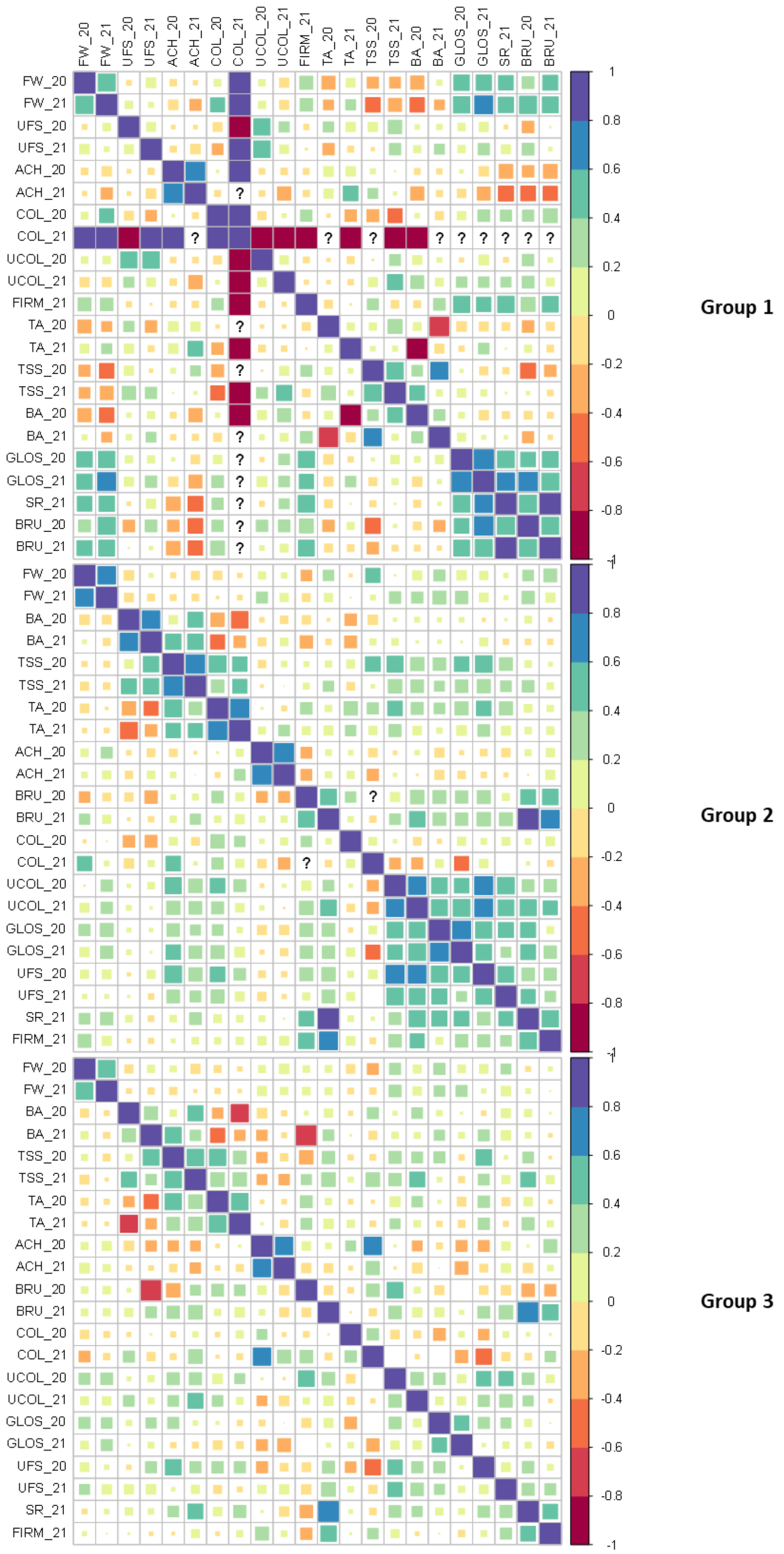

Supplementary Figure S8. Correlations between the 12 traits for each year for each genetic group. '?' when correlation could not be calculated.

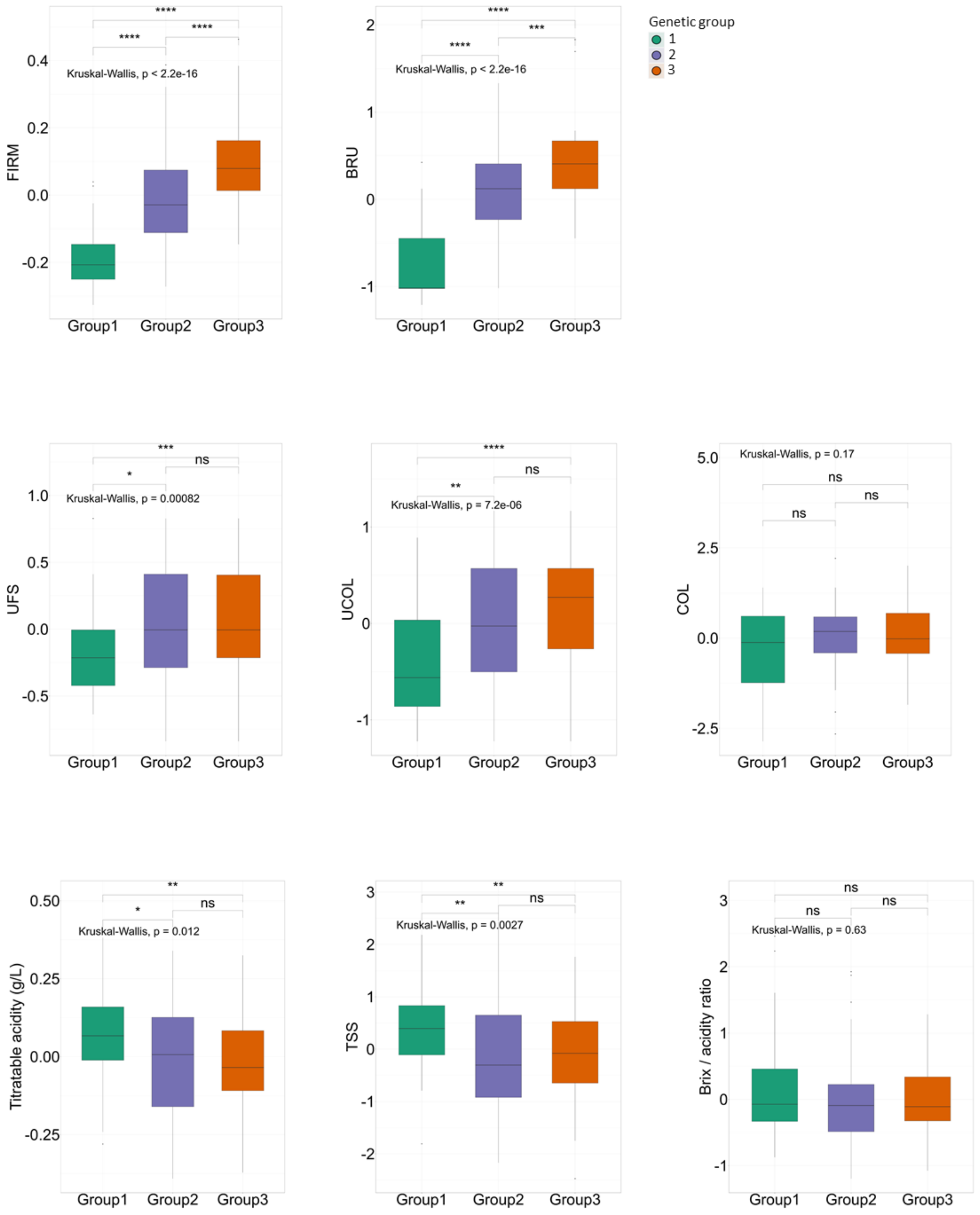

Supplementary Figure S9. Comparisons of 2-year BLUP values for FIRM, BRU, UFS, UCOL, COL, TA, TSS and BA among genetic groups. Genetic groups 1, 2 and 3 are colored in green, purple and orange, respectively. UFS, uniformity of fruit shape; COL, skin color; UCOL, uniformity of skin color; FIRM, firmness; TA, titratable acidity; TSS, total soluble solids; BRU, bruisedness.

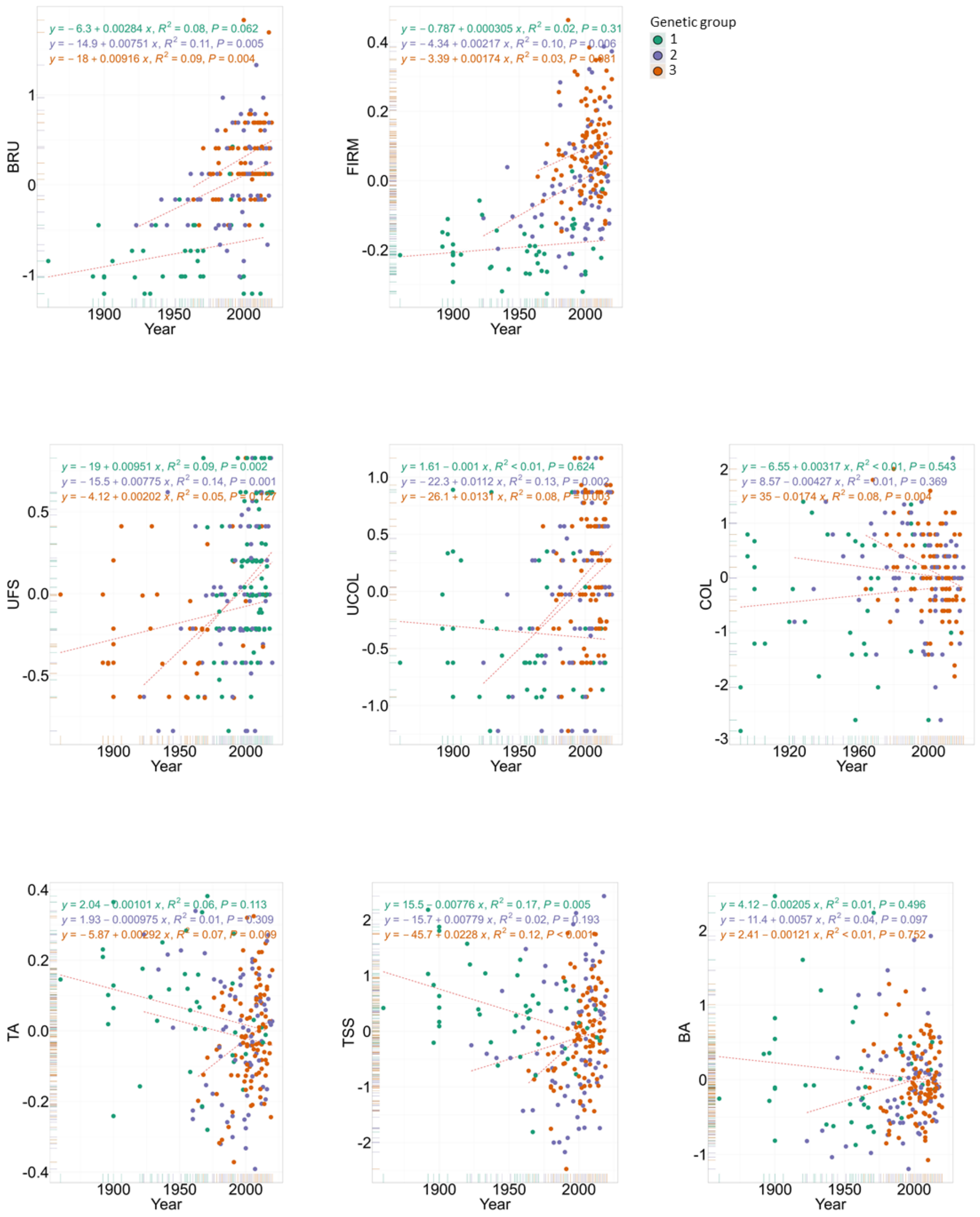

Supplementary Figure S10. Genetic gains for FIRM, BRU, UFS, UCOL, TA, TSS and BA among genetic groups. Genetic groups 1, 2 and 3 are colored in green, purple and orange, respectively. UFS, uniformity of fruit shape; COL, skin color; UCOL, uniformity of skin color; FIRM, firmness; TA, titratable acidity; TSS, total soluble solids; BRU, bruisedness.

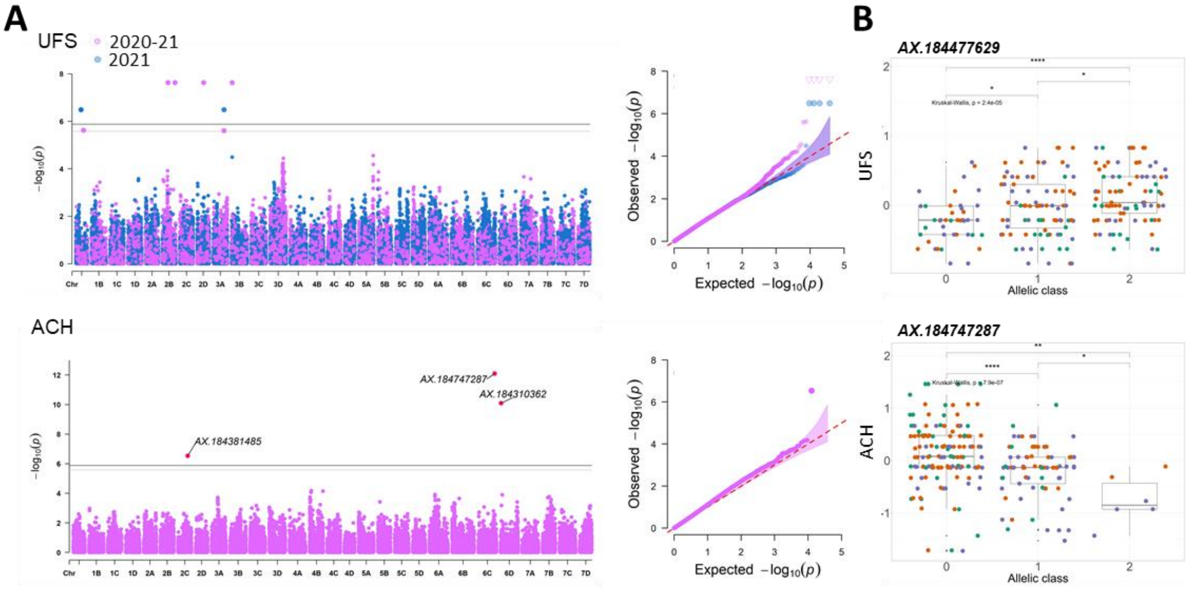

Supplementary Figure S11. Genome wide association of study of UFS and ACH. (A) Manhattan and Q-Q plots for yearly and 2-year BLUP values. (B) Effect of the most significant SNP markers. Genetic groups 1, 2 and 3 are colored in green, purple and orange, respectively. UFS, uniformity of fruit shape; ACH, position and depth of achenes. Marker classes are as follows: 0=AA genotype, 1=AB, and 2=BB genotype according to the Axiom™ Strawberry FanaSNP 50k.

Supplementary Table S1. List of the 223 genotypes. Origin (country/state), Continent, Year of release, group of structure and their estimated membership fractions (%). Groups were identified using Structure. Origins and dates of release/observation from Cost836, GenBerry database, CPOV, UC Davis.

					Estimated membership fractions		
Name	Origin1	Origin2	Year	Group	Group 1	Group 2	Group 3
Akicheme	Japan	Asia	1992	Heirloom & related	1.00	0.00	0.00
Anablanca	France	Europe	2012	Heirloom & related	1.00	0.00	0.00
Avalon_ex	Univ.Calif.	America	1998	Heirloom & related	0.86	0.08	0.06
Belle_et_Bonne	France	Europe	1958	Heirloom & related	0.83	0.17	0.00
Blanche_ananas	Germany	Europe	1900	Heirloom & related	1.00	0.00	0.00
Blanche_du_Morvan	France	Europe	2000	Heirloom & related	1.00	0.00	0.00
CF0964	France	Europe	1991	Heirloom & related	0.69	0.16	0.16
Darsidor	France	Europe	1992	Heirloom & related	0.36	0.34	0.30
F_Eure_et_Loire	France	Europe	2000	Heirloom & related	1.00	0.00	0.00
Fertilite	France	Europe	1900	Heirloom & related	0.66	0.34	0.00
Frel	UK	Europe	1990	Heirloom & related	0.99	0.00	0.01
Gento	Germany	Europe	1967	Heirloom & related	0.56	0.33	0.11
Grande	Germany	Europe	1967	Heirloom & related	0.64	0.29	0.06
Great_Blossom	NA	NA	1900	Heirloom & related	0.91	0.01	0.09
Hative_de_Caen	France	Europe	1928	Heirloom & related	0.90	0.10	0.00
Haveland	Germany	Europe	1971	Heirloom & related	0.84	0.16	0.00
Jive	Netherlands	Europe	2015	Heirloom & related	1.00	0.00	0.00
Josif_Mahommet	Ukraine	Europe	1900	Heirloom & related	0.94	0.04	0.02
Liberation_d'Orleans	Canada	America	1962	Heirloom & related	0.81	0.15	0.03
Louis_Gauthier	France	Europe	1896	Heirloom & related	0.54	0.46	0.00
Madame_Lefebvre	France	Europe	1900	Heirloom & related	0.72	0.24	0.03
Madame_Moutot	France	Europe	1906	Heirloom & related	0.91	0.01	0.08
Mara des Bois	France	Europe	1991	Heirloom & related	0.48	0.23	0.30
Marie_France	France	Europe	1955	Heirloom & related	0.90	0.05	0.04
Merton_Ruby	UK	Europe	1965	Heirloom & related	0.44	0.13	0.43
Mieze_Schiendler	Germany	Europe	1933	Heirloom & related	0.95	0.03	0.01
Mount_Everest	France	Europe	1971	Heirloom & related	0.88	0.09	0.02
Mysowka	Russia	Europe	1958	Heirloom & related	0.76	0.16	0.08
Ostara	Netherlands	Europe	1969	Heirloom & related	0.64	0.27	0.09
Primek	Denmark	Europe	1990	Heirloom & related	0.63	0.31	0.06
Rabunda	Netherlands	Europe	1964	Heirloom & related	0.72	0.26	0.02
Reiko	Japan	Asia	1990	Heirloom & related	0.45	0.32	0.23
Revada	Netherlands	Europe	1956	Heirloom & related	0.89	0.11	0.00
RG084	Chile	America	NA	Heirloom & related	1.00	0.00	0.00
RG085	Chile	America	NA	Heirloom & related	0.79	0.21	0.00
Rheingold	Germany	Europe	1920	Heirloom & related	0.43	0.37	0.20
Royal_Sovereign	UK	Europe	1892	Heirloom & related	0.94	0.00	0.06
Saint_Joseph	France	Europe	1892	Heirloom & related	0.62	0.34	0.04
Sans_Rivale	France	Europe	1937	Heirloom & related	0.99	0.00	0.01
Segaline	France	Europe	1900	Heirloom & related	1.00	0.00	0.00
Senga_Sengana	Germany	Europe	1954	Heirloom & related	0.59	0.31	0.11
Souvenir_de_Charles_Machiroux	Belgium	Europe	1942	Heirloom & related	0.89	0.11	0.00
Spate_Leopold	Germany	Europe	1922	Heirloom & related	0.91	0.00	0.09
St_Antoine_de_Padoue	France	Europe	1896	Heirloom & related	0.77	0.21	0.02
Toscana	Netherlands	Europe	2013	Heirloom & related	0.40	0.28	0.32
Vigerla	Germany	Europe	1963	Heirloom & related	0.51	0.44	0.05
Ville_de_Caen	France	Europe	1922	Heirloom & related	0.83	0.08	0.10
Ville_de_Paris	France	Europe	1929	Heirloom & related	0.84	0.16	0.00
White_Pine	USA	America	1860	Heirloom & related	0.89	0.05	0.05
Addie	Italy	Europe	1982	European mixed	0.26	0.61	0.12
Allstar	Maryland	America	1981	European mixed	0.09	0.86	0.05
AN100851	Italy	Europe	2020	European mixed	0.02	0.59	0.39
Anais	France	Europe	2008	European mixed	0.24	0.53	0.23
Arking	Maryland	America	1981	European mixed	0.16	0.77	0.07
Bavo	Belgium	Europe	2000	European mixed	0.47	0.53	0.00
Belrubi	France	Europe	1962	European mixed	0.04	0.96	0.00
Blackmore	Maryland	America	1923	European mixed	0.13	0.87	0.00
Candiss	France	Europe	2008	European mixed	0.03	0.73	0.23
Catskill	New-York	America	1934	European mixed	0.34	0.54	0.12
CF11322	France	Europe	2018	European mixed	0.00	0.65	0.35
CF11A91	France	Europe	2018	European mixed	0.17	0.48	0.34
CF12074	France	Europe	2019	European mixed	0.02	0.63	0.34

Table S1 - to be continued

CF2036	France	Europe	2004	European mixed	0.06	0.63	0.31
CF2299	France	Europe	2005	European mixed	0.00	0.63	0.37
CF2314	France	Europe	2005	European mixed	0.14	0.84	0.01
CF2337	France	Europe	2005	European mixed	0.02	0.93	0.05
CF2725	France	Europe	2007	European mixed	0.34	0.41	0.25
CF2923	France	Europe	2009	European mixed	0.00	0.58	0.42
CF3008	France	Europe	2010	European mixed	0.00	0.82	0.18
CF7135	France	Europe	2012	European mixed	0.00	0.59	0.40
Christine	Netherlands	Europe	2002	European mixed	0.04	0.65	0.30
Ciflorette	France	Europe	1998	European mixed	0.03	0.79	0.18
Cigaline	France	Europe	1996	European mixed	0.02	0.98	0.00
Cigoulette	France	Europe	1996	European mixed	0.01	0.63	0.37
Ciloe	France	Europe	1998	European mixed	0.05	0.95	0.00
Clery	Italy	Europe	2002	European mixed	0.00	0.63	0.36
Cristina	Italy	Europe	2013	European mixed	0.11	0.88	0.02
Darlisette	France	Europe	2004	European mixed	0.06	0.71	0.23
Darselect	France	Europe	1996	European mixed	0.00	0.54	0.46
Dely	France	Europe	2011	European mixed	0.00	0.67	0.33
Dipred	Italy	Europe	2014	European mixed	0.03	0.53	0.44
Donner	Univ.Calif.	America	1945	European mixed	0.14	0.60	0.26
Dukat	Poland	Europe	1985	European mixed	0.36	0.62	0.02
Earlyglow	Maryland	America	1975	European mixed	0.00	0.99	0.01
Elegance	UK	Europe	2008	European mixed	0.16	0.70	0.14
Elsanta	Netherlands	Europe	1981	European mixed	0.00	1.00	0.00
Elsinore	Italy	Europe	1970	European mixed	0.05	0.50	0.45
Elvira	Netherlands	Europe	1967	European mixed	0.39	0.61	0.00
Frida	Norway	Europe	2001	European mixed	0.09	0.65	0.26
Gariguette	France	Europe	1976	European mixed	0.13	0.85	0.01
Georg_Soltwedel	Germany	Europe	1941	European mixed	0.17	0.71	0.12
Gerida	Switzerland	Europe	1990	European mixed	0.15	0.85	0.00
Gladis	France	Europe	2015	European mixed	0.20	0.74	0.06
Gorella	Netherlands	Europe	1960	European mixed	0.06	0.94	0.00
Guillerette	France	Europe	2010	European mixed	0.01	0.51	0.48
Jewel	New-York	America	1985	European mixed	0.21	0.79	0.00
Linn	Maryland	America	1969	European mixed	0.37	0.50	0.13
Magnum	France	Europe	2016	European mixed	0.14	0.65	0.20
Mailing_Pearl	UK	Europe	2012	European mixed	0.00	0.54	0.46
Manille	France	Europe	2005	European mixed	0.37	0.38	0.25
Maxim	Belgium	Europe	1986	European mixed	0.34	0.66	0.00
Morioka	Japan	Asia	1960	European mixed	0.21	0.67	0.12
Nyoho	Japan	Asia	1984	European mixed	0.32	0.43	0.25
Osiris	France	Europe	2017	European mixed	0.02	0.50	0.48
Patty	Italy	Europe	1999	European mixed	0.08	0.84	0.08
Pegasus	UK	Europe	1990	European mixed	0.29	0.71	0.00
Queen_Elisa	Italy	Europe	2003	European mixed	0.10	0.86	0.04
Renaissance	Germany	Europe	2017	European mixed	0.27	0.54	0.19
Rubis_Des_Jardins	France	Europe	2016	European mixed	0.00	0.51	0.49
Sara	Sweden	Europe	1988	European mixed	0.46	0.48	0.06
Sonata	Netherlands	Europe	2005	European mixed	0.09	0.91	0.00
Sophie	UK	Europe	1997	European mixed	0.46	0.49	0.05
St_Jean_d'Orleans	Canada	America	2004	European mixed	0.31	0.63	0.05
St_Laurent_d'Orleans	Canada	America	2004	European mixed	0.20	0.75	0.05
Surecrop	Maryland	America	1951	European mixed	0.15	0.85	0.00
Sweede	Univ.Calif.	America	1980	European mixed	0.31	0.37	0.32
Tea	Italy	Europe	2015	European mixed	0.02	0.63	0.35
Tecla	Italy	Europe	2000	European mixed	0.37	0.63	0.00
Tochiotone	Japan	Asia	1996	European mixed	0.28	0.44	0.28
US_292	Maryland	America	1992	European mixed	0.18	0.76	0.06
US_438	Maryland	America	1992	European mixed	0.15	0.68	0.18
Valeta	Netherlands	Europe	1983	European mixed	0.08	0.92	0.00
Vidoca	Netherlands	Europe	1988	European mixed	0.07	0.81	0.12
White_Gorella	Italy	Europe	2000	European mixed	0.08	0.92	0.00

Table S1 - to be continued

Aghate	Florida	America	1992	American & European mixed	0.00	0.33	0.67
Alba	Italy	Europe	2002	American & European mixed	0.00	0.45	0.55
Albion	Univ.Calif.	USA	2004	American & European mixed	0.00	0.00	1.00
Altesse	NA	NA	2019	American & European mixed	0.15	0.20	0.65
Amandine	France	Europe	2012	American & European mixed	0.19	0.00	0.81
AN104521	Italy	Europe	2020	American & European mixed	0.00	0.33	0.67
Anabelle	France	Europe	2003	American & European mixed	0.24	0.15	0.61
Angelina	France	Europe	1998	American & European mixed	0.04	0.39	0.57
Aprica	Italy	Europe	2018	American & European mixed	0.00	0.49	0.51
Armelle	France	Europe	2018	American & European mixed	0.00	0.14	0.86
Aromas	Univ.Calif.	America	1997	American & European mixed	0.00	0.00	1.00
Asia	Italy	Europe	2005	American & European mixed	0.02	0.43	0.55
Ava	UK	Europe	2001	American & European mixed	0.31	0.12	0.57
Bogota	Netherlands	Europe	1971	American & European mixed	0.31	0.04	0.65
Brighton	Univ.Calif.	America	1978	American & European mixed	0.15	0.10	0.75
Camarosa	Univ Calif	USA	1992	American & European mixed	0.00	0.00	1.00
Candonga	Spain	Europe	2003	American & European mixed	0.00	0.00	1.00
Capella_1	France	Europe	2001	American & European mixed	0.06	0.15	0.79
Capitola	Univ.Calif.	America	1989	American & European mixed	0.10	0.03	0.86
Capris	France	Europe	2010	American & European mixed	0.00	0.42	0.58
CF0129	France	Europe	1989	American & European mixed	0.00	0.43	0.57
CF1116	France	Europe	1992	American & European mixed	0.00	0.31	0.69
CF2070	France	Europe	2004	American & European mixed	0.01	0.47	0.52
CF2419	France	Europe	2005	American & European mixed	0.37	0.18	0.45
CF2443	France	Europe	2005	American & European mixed	0.23	0.06	0.71
CF2845	France	Europe	2008	American & European mixed	0.00	0.47	0.52
CF2884	France	Europe	2008	American & European mixed	0.03	0.39	0.58
CF3106	France	Europe	2010	American & European mixed	0.23	0.01	0.76
CF3107	France	Europe	2010	American & European mixed	0.00	0.37	0.63
CF4503	France	Europe	2010	American & European mixed	0.04	0.35	0.61
CF6923	France	Europe	2010	American & European mixed	0.12	0.25	0.62
CF7025	France	Europe	2011	American & European mixed	0.00	0.35	0.65
CF7026	France	Europe	2011	American & European mixed	0.05	0.28	0.67
CF7127	France	Europe	2012	American & European mixed	0.05	0.24	0.70
CF9041	France	Europe	2015	American & European mixed	0.00	0.45	0.55
CF9335	France	Europe	2015	American & European mixed	0.08	0.38	0.54
CF9838	France	Europe	2015	American & European mixed	0.12	0.35	0.53
Chandler	Univ.Calif.	America	1983	American & European mixed	0.00	0.00	1.00
Chapelaine	France	Europe	2000	American & European mixed	0.12	0.16	0.71
Cifrance	France	Europe	1996	American & European mixed	0.00	0.48	0.52
Cigaëlle	France	Europe	1999	American & European mixed	0.14	0.32	0.54
Cijosée	France	Europe	2001	American & European mixed	0.25	0.04	0.72
Cir_104	France	Europe	2010	American & European mixed	0.20	0.07	0.72
Cir_107	France	Europe	2010	American & European mixed	0.00	0.05	0.95
CIR_111	France	Europe	2011	American & European mixed	0.00	0.34	0.66
Cir_112	France	Europe	2011	American & European mixed	0.21	0.14	0.65
Cir_121	France	Europe	2012	American & European mixed	0.00	0.47	0.53
cir_129	France	Europe	2012	American & European mixed	0.23	0.08	0.69
Cir_146	France	Europe	2014	American & European mixed	0.00	0.26	0.74
Cir_147_	France	Europe	2014	American & European mixed	0.22	0.01	0.78
Cir_159	France	Europe	2015	American & European mixed	0.02	0.29	0.70
Cirafine	France	Europe	1997	American & European mixed	0.25	0.02	0.73
Cirano	France	Europe	1997	American & European mixed	0.28	0.15	0.57
Cireine	France	Europe	1996	American & European mixed	0.00	0.44	0.56
Deluxe	France	Europe	2012	American & European mixed	0.00	0.43	0.57
Diademe	France	Europe	2016	American & European mixed	0.00	0.38	0.62
Divine	France	Europe	2007	American & European mixed	0.00	0.46	0.54
Dona	France	Europe	2006	American & European mixed	0.02	0.21	0.77
Dover	Florida	America	1980	American & European mixed	0.14	0.39	0.47
DPI_Rubygem	Australia	Australia	2004	American & European mixed	0.00	0.08	0.92
Dream	France	Europe	2012	American & European mixed	0.04	0.22	0.74
Elianny	Netherlands	Europe	1998	American & European mixed	0.00	0.42	0.58
Evie3	UK	Europe	2006	American & European mixed	0.12	0.10	0.78
Favette	France	Europe	1976	American & European mixed	0.22	0.37	0.41

Table S1 - to be continued

Festival	Florida	America	2000	American & European mixed	0.00	0.04	0.96
Flavia	Italy	Europe	2018	American & European mixed	0.00	0.04	0.96
FR028	France	Europe	2008	American & European mixed	0.09	0.13	0.78
FR179	France	Europe	2016	American & European mixed	0.21	0.06	0.73
Gardena	Italy	Europe	1991	American & European mixed	0.25	0.37	0.38
Gustine	France	Europe	2009	American & European mixed	0.12	0.43	0.46
Kilo	Italy	Europe	2001	American & European mixed	0.00	0.04	0.96
Linosa	Italy	Europe	2011	American & European mixed	0.01	0.15	0.84
Malling_Centenary	UK	Europe	2013	American & European mixed	0.09	0.18	0.72
Monterey	Univ.Calif.	America	2008	American & European mixed	0.02	0.00	0.98
Murano	Italy	Europe	2015	American & European mixed	0.00	0.47	0.53
Naiad	Italy	Europe	2000	American & European mixed	0.04	0.03	0.93
Nora	Italy	Europe	2001	American & European mixed	0.01	0.12	0.87
Oso_Grande	Univ.Calif.	America	1987	American & European mixed	0.00	0.00	1.00
Pajaro	Univ.Calif.	America	1978	American & European mixed	0.01	0.17	0.82
Palatina	Italy	Europe	1997	American & European mixed	0.00	0.17	0.83
Polka	Netherlands	Europe	1980	American & European mixed	0.00	0.00	1.00
Portola	Univ.Calif.	America	2008	American & European mixed	0.06	0.00	0.94
Primy	NA	NA	1990	American & European mixed	0.00	0.30	0.70
Rosalinda	Florida	America	1999	American & European mixed	0.03	0.21	0.76
Saint_Pierre	Canada	America	2004	American & European mixed	0.07	0.39	0.53
San_Andreas	Univ.Calif.	America	2008	American & European mixed	0.02	0.00	0.98
Santana	Univ.Calif.	America	1982	American & European mixed	0.16	0.37	0.48
Sasha	UK	Europe	2008	American & European mixed	0.00	0.42	0.58
Scott	USA	America	1980	American & European mixed	0.14	0.09	0.77
Sequoia	Univ.Calif.	America	1968	American & European mixed	0.12	0.26	0.62
Siabelle	NA	NA	2001	American & European mixed	0.28	0.19	0.54
Sibilla	Italy	Europe	2016	American & European mixed	0.00	0.41	0.59
Soquel	Univ.Calif.	America	1982	American & European mixed	0.22	0.00	0.78
Sweet_Ann	California	America	2012	American & European mixed	0.03	0.00	0.97
Tioga	Univ.Calif.	America	1964	American & European mixed	0.18	0.09	0.73
Tustin	Univ.Calif.	America	1982	American & European mixed	0.06	0.01	0.93
Ventana	Univ.Calif.	America	2001	American & European mixed	0.00	0.00	1.00
Virtue	Netherlands	Europe	2007	American & European mixed	0.09	0.00	0.91
Vivara	Italy	Europe	2014	American & European mixed	0.00	0.20	0.80

Supplementary Table S2. Pearson correlations between 2-year estimated BLUP values for the 12 traits.

Trait 1	Trait 2	Pearson correlation	p-value
Skin Resistance	Bruiseness	0.87	0
Firmness	Bruiseness	0.73	0
Firmness	Skin Resistance	0.72	0
Color Homogeneity	Shape Homogeneity	0.61	0
Bruiseness	Glossiness	0.60	0
Mean Weight	Glossiness	0.58	0
Skin Resistance	Glossiness	0.58	0
Mean Weight	Firmness	0.54	0
Mean Weight	Skin Resistance	0.51	1.51E-14
Firmness	Glossiness	0.50	1.91E-14
Mean Weight	Bruiseness	0.50	2.65E-13
Glossiness	Color Homogeneity	0.47	1.20E-12
Titratable Acidity	Total Soluble Solids	0.44	2.03E-11
Glossiness	Shape Homogeneity	0.44	5.23E-11
Bruiseness	Color Homogeneity	0.41	1.28E-09
Skin Resistance	Color Homogeneity	0.40	1.53E-09
Skin Resistance	Shape Homogeneity	0.40	1.91E-09
Bruiseness	Shape Homogeneity	0.39	1.30E-08
Mean Weight	Color Homogeneity	0.36	6.51E-08
Firmness	Color Homogeneity	0.35	1.22E-07
Firmness	Shape Homogeneity	0.34	3.88E-07
Mean Weight	Shape Homogeneity	0.29	2.29E-05
Shape Homogeneity	Total Soluble Solids	0.27	6.26E-05
Color Homogeneity	Total Soluble Solids	0.26	9.73E-05
Firmness	External Color	0.23	0.0008
Skin Resistance	External Color	0.20	0.00474
Mean Weight	External Color	0.17	0.01721
Bruiseness	External Color	0.16	0.02319
Achene Insertion	Titratable Acidity	0.11	0.12552
Glossiness	External Color	0.10	0.15265
Color Homogeneity	Titratable Acidity	0.10	0.1543
Shape Homogeneity	Titratable Acidity	0.09	0.17076
Skin Resistance	Total Soluble Solids	0.05	0.49268
Firmness	Achene Insertion	0.04	0.54422
Mean Weight	Achene Insertion	0.01	0.87498
Glossiness	Total Soluble Solids	0.01	0.91485
Bruiseness	Total Soluble Solids	0.00	0.95612
Achene Insertion	External Color	-0.01	0.88308
Skin Resistance	Titratable Acidity	-0.01	0.83638
Achene Insertion	Total Soluble Solids	-0.02	0.74097
Shape Homogeneity	External Color	-0.02	0.73203
Titratable Acidity	External Color	-0.03	0.67376
Color Homogeneity	External Color	-0.05	0.5183
Bruiseness	Titratable Acidity	-0.05	0.48536
Firmness	Total Soluble Solids	-0.07	0.29334
Firmness	Titratable Acidity	-0.08	0.23537
Achene Insertion	Color Homogeneity	-0.09	0.1992
Achene Insertion	Shape Homogeneity	-0.09	0.18106
Glossiness	Titratable Acidity	-0.11	0.13446
Total Soluble Solids	External Color	-0.12	0.08283
Bruiseness	Achene Insertion	-0.14	0.04316
Skin Resistance	Achene Insertion	-0.14	0.0373
Mean Weight	Titratable Acidity	-0.17	0.01697
Glossiness	Achene Insertion	-0.21	0.00234
Mean Weight	Total Soluble Solids	-0.23	0.00088

Supplementary Table S3. List of significant trait associations obtained for the GWAS on the 12 traits. Position Camarosa and Position Royal Royce: physical positions on Camarosa and Royal Royce reference genomes. MAF, minor allele frequency; Phenotypic variance explained (%) calculated by GAPIT3[80].

Trait	Year	SNP	Chromosome	Position Camarosa	Position Royal Royce	P.value	Effect	maf	PVE
Fruit weight (FW)	combined values	AX-184413183	1B	19 119 571	15 971 709	6.74E-09	1.3	0.37	5.5
	combined values	AX-184592155	2D	15 565 564	8 801 569	3.27E-12	1.8	0.33	11.8
	combined values	AX-184241601	5B	17 045 086	10 918 733	1.60E-07	-1.5	0.21	3.6
Shape homogeneity (UFS)	2021	AX-184611387	1A	9 927 298	10 221 629	3.24E-07	0.5	0.21	0.8
	2021	AX-184554177	1A	10 002 081	10 295 193	3.24E-07	2.0	0.21	0.7
	2021	AX-89904139	1A	9 957 207	--	3.24E-07	7.3	0.21	2.0
	2021	AX-184458801	3A	21 213 134	9 650 845	3.24E-07	-0.8	0.18	8.8
	Combined values	AX-184043005	2B	11 149 178	11 940 989	2.37E-08	-0.3	0.08	6.0
	Combined values	AX-184880676	2B	25 135 835	25 837 267	2.37E-08	-0.2	0.26	3.7
	Combined values	AX-184466777	2D	12 871 717	11 774 978	2.37E-08	0.0	0.08	0.0
	Combined values	AX-184477629	3B	1 541 393	1 842 542	2.37E-08	0.1	0.40	4.8
Achene position (ACH)	Combined values	AX-184381485	2C	19 190 988	7 710 274	2.90E-07	-0.3	0.34	6.3
	Combined values	AX-184747287	6C	32 305 891	30 352 992	8.06E-13	-0.3	0.20	12.4
	Combined values	AX-184310362	6D	939 082	32 008 538	8.06E-11	0.4	0.20	16.3
Skin color (COL)	2020	AX-184965421	5D	14 022 053	13 542 145	8.68E-09	-0.7	0.41	23.4
	Combined values	AX-166514401	5C	11 987 143	--	1.46E-07	0.5	0.28	1.7
	Combined values	AX-184965421	5D	14 022 053	13 542 145	1.01E-08	-0.5	0.42	3.8
	Combined values	AX-184965919	6A	14 476 176	21 070 121	1.17E-07	0.4	0.23	2.6
	Combined values	AX-184300087	7B	1 386 945	23 020 879	1.01E-10	-0.4	0.22	3.0
	Combined values	AX-184090167	7C	19 438 028	13 528 856	2.92E-07	-0.6	0.11	9.9
Firmness (FIRM)	2021	AX-184521799	2A	17 814 521	5 400 298	7.69E-08	0.0	0.29	4.4
	2021	AX-123522331	2C	22 908 001	3 685 634	1.33E-06	0.3		NA
	2021	AX-184477554	3D	29 275 014	2 454 715	6.07E-12	0.1	0.46	11.2
	2021	AX-184039356	6A	7 277 130	27 533 782	1.02E-08	0.0	0.38	6.3
Titratable acidity (TA)	2020	AX-184091372	1A	13 540 517	13 469 152	2.30E-12	0.1	0.30	17.1
	2020	AX-184457703	3D	30 090 404	--	4.22E-09	-0.1	0.21	17.9
	Combined values	AX-166514185	5C	6 983 698	8 203 430	1.06E-06	-0.1	0.36	3.5
	Combined values	AX-184595531	6A	25 621 066	9 172 070	1.55E-08	0.1	0.15	35.3
	Combined values	AX-184462338	6C	27 458 040	--	8.47E-08	-0.1	0.34	6.8
TSS (Brix)	2020	AX-184131652	1B	4 174 690	1 302 571	4.64E-07	-0.4	0.13	3.6
	2020	AX-184718481	2D	13 298 291	--	4.64E-07	0.4	0.40	1.4
	2020	AX-184477629	3B	1 541 393	1 842 542	4.64E-07	0.3	0.40	0.7
	2020	AX-184970304	5A	12 400 711	10 544 768	4.64E-07	-0.4	0.14	0.9
	2020	AX-184507945	5A	21 971 093	18 609 839	4.64E-07	0.3	0.20	0.0
	2020	AX-184330352	5A	22 141 939	18 780 692	4.64E-07	0.0	0.20	0.0
	2020	AX-184219801	5A	22 266 307	18 905 072	4.64E-07	0.3	0.20	0.0
	2020	AX-184418966	5A	24 950 308	21 580 601	4.64E-07	0.1	0.33	0.2
	2020	AX-184355355	6B	27 647 943	10 765 128	4.64E-07	-0.5	0.16	1.4
	2020	AX-184221848	6C	33 293 635	29 374 007	4.64E-07	0.3	0.38	0.6
	2020	AX-184920058	7B	4 515 979	19 984 301	4.64E-07	0.4	0.50	1.6
	2020	AX-184079508	7C	26 901 718	19 354 380	4.64E-07	-0.3	0.30	0.5
	2021	AX-184399755	6B	31 578 303	9 217 798	3.24E-10	1.3	0.13	19.0
	2021	AX-184047575	7C	20 267 768	--	7.27E-08	-0.8	0.24	7.7
	Combined values	AX-184505625	3B	4 434 681	4 139 234	1.62E-08	0.3	0.35	5.3
	Combined values	AX-184970304	5A	12 400 711	10 544 768	1.62E-08	-0.2	0.14	2.1
	Combined values	AX-184179821	5A	22 384 778	18 999 088	1.62E-08	-0.2	0.21	2.8
	Combined values	AX-184864732	6D	12 013 420	9 512 125	1.62E-08	-0.3	0.33	4.8
	Combined values	AX-184920058	7B	4 515 979	19 984 301	1.62E-08	0.3	0.50	4.0
	Combined values	AX-184560339	7C	19 112 988	13 209 597	1.62E-08	0.4	0.26	3.4
BA ratio	2021	AX-184282016	3B	4 095 262	3 825 622	3.62E-07	-0.5	0.09	11.4
	2021	AX-184052133	3B	4 097 709	3 828 069	3.62E-07	0.3	0.17	1.6
	2021	AX-184452909	6D	1 646 690	32 715 821	3.62E-07	0.5	0.19	5.0
	Combined values	AX-184399755	6B	31 578 303	9 217 798	7.88E-13	0.5	0.13	65.8
Glossiness (GLO)	2020	AX-184177060	3D	27 845 440	3 815 908	2.77E-11	-0.7	0.21	28.7
	2020	AX-184352835	5A	28 239 789	24 966 878	4.80E-07	-0.4	0.28	11.1
	2021	AX-184494194	1C	11 470 545	10 966 861	2.43E-07	0.4	0.20	17.8
	2021	AX-184177060	3D	27 845 440	3 815 908	5.49E-13	-0.7	0.24	26.3
	combined values	AX-184599570	3D	26 901 693	4 775 280	9.01E-08	0.4	0.22	4.7
	combined values	AX-184177060	3D	27 845 440	3 815 908	9.01E-10	-0.8	0.23	26.2
	combined values	AX-184951955	4C	727 143	26 073 661	1.15E-06	-0.3	0.37	3.4
	combined values	AX-184408294	5A	28 658 239	25 341 723	2.54E-07	-0.3	0.20	4.1
Skin resistance (SR)	2021	AX-184177060	3D	27 845 440	3 815 908	6.45E-07	-0.5	0.24	8.4
	2021	AX-184127736	4A	20 012 930	16 433 479	6.45E-07	0.3	0.19	5.4
	2021	AX-184230747	5B	15 853 776	12 173 853	6.45E-07	-0.3	0.20	3.6
	2021	AX-184130926	7A	25 616 815	18 667 225	6.45E-07	0.3	0.31	7.3
	2021	AX-123359788	7A	28 581 204	--	6.45E-07	-0.2	0.32	1.6
Bruiseness (BRU)	combined values	AX-184940044	1A	3 531 536	--	1.38E-07	-0.2	0.30	6.9
	combined values	AX-184203769	1B	9 439 391	7 276 991	2.13E-12	-0.2	0.49	6.9
	combined values	AX-184408176	5A	18 814 640	16 414 924	2.59E-08	0.2	0.10	4.4
	combined values	AX-184399480	6B	5 731 875	30 532 752	1.37E-09	-0.3	0.21	5.5
	combined values	AX-184713611	7C	20 943 204	15 149 370	8.36E-07	-0.2	0.33	2.8

Supplementary Table S4. Genome scan outputs for the 71 trait associations. Position Camarosa and Position Royal Royce: physical positions on Camarosa and Royal Royce reference genomes. pval_Mahalanobis, p-values of the genome scan based on Mahalanobis distance. threshold, p-value thresholds at 0.01 (**) and 0.05 (*). π_group1, π_group2, π_group3, π values of the genome scans performed on respectively genetic groups 1, 2 and 3 within 400kb windows.

Trait	Year	SNP	Chromosome	Position Camarosa	Position Royal Royce	pval_Mahalanobis	threshold	π_group1	π_group2	π_group3
Glossiness (GLO)	2021	AX-184177060	3D	27845440	3815908	0.001	**	0.44	0.32	0.15
Glossiness (GLO)	2020	AX-184177060	3D	27845440	3815908	0.001	**	0.44	0.32	0.15
Glossiness (GLO)	combinedvalues	AX-184177060	3D	27845440	3815908	0.001	**	0.44	0.32	0.15
Skin resistance (SR)	2021	AX-184177060	3D	27845440	3815908	0.001	**	0.44	0.32	0.15
Firmness (FIRM)	2021	AX-184477554	3D	29275014	2454715	0.008	**	0.40	0.39	0.29
Shape homogeneity (UFS)	Combinedvalues	AX-184880676	2B	25135835	25837267	0.008	**	0.36	0.30	0.31
Glossiness (GLO)	2020	AX-184352835	5A	28239789	24966878	0.031	*	0.35	0.34	0.40
Skin resistance (SR)	2021	AX-184130926	7A	25616815	18667225	0.032	*	0.31	0.35	0.35
TSS (Brix)	Combinedvalues	AX-184864732	6D	12013420	9512125	0.041	*	0.36	0.43	0.22
Titratable acidity (TA)	2020	AX-184457703	3D	30090404	--	0.066		0.37	0.40	0.34
Skin color (COL)	Combinedvalues	AX-184300087	7B	1386945	23020879	0.084		0.34	0.33	0.30
Achene position (ACH)	Combinedvalues	AX-184381485	2C	19190988	7710274	0.088		0.39	0.36	0.37
TSS (Brix)	Combinedvalues	AX-184505625	3B	4434681	4139234	0.138		0.39	0.44	0.39
Skin resistance (SR)	2021	AX-123359788	7A	28581204	--	0.139		0.31	0.34	0.34
TSS (Brix)	Combinedvalues	AX-184920058	7B	4515979	19984301	0.179		0.41	0.43	0.30
TSS (Brix)	2020	AX-184920058	7B	4515979	19984301	0.179		0.41	0.43	0.30
TSS (Brix)	Combinedvalues	AX-184560339	7C	19112988	13209597	0.234		0.42	0.34	0.35
Titratable acidity (TA)	Combinedvalues	AX-166514185	5C	6983698	8203430	0.238		0.34	0.37	0.34
Glossiness (GLO)	2021	AX-184494194	1C	11470545	10966861	0.253		0.43	0.42	0.18
Titratable acidity (TA)	Combinedvalues	AX-184595531	6A	25621066	9172070	0.301		0.36	0.37	0.41
Achene position (ACH)	Combinedvalues	AX-184310362	6D	939082	32008538	0.31		0.38	0.40	0.30
TSS (Brix)	2020	AX-184355355	6B	27647943	10765128	0.313		0.37	0.34	0.34
Skin color (COL)	Combinedvalues	AX-166514401	5C	11987143	--	0.351		0.35	0.37	0.39
TSS (Brix)	2020	AX-184131652	1B	4174690	1302571	0.358		0.25	0.36	0.39
Bruiseness (BRU)	combinedvalues	AX-184399480	6B	5731875	30532752	0.364		0.41	0.33	0.43
Glossiness (GLO)	combinedvalues	AX-184408294	5A	28658239	25341723	0.39		0.35	0.36	0.44
Achene position (ACH)	Combinedvalues	AX-184747287	6C	32305891	30352992	0.393		0.42	0.45	0.38
TSS (Brix)	Combinedvalues	AX-184970304	5A	12400711	10544768	0.396		0.32	0.41	0.36
TSS (Brix)	2020	AX-184970304	5A	12400711	10544768	0.396		0.32	0.41	0.36
Shape homogeneity (UFS)	Combinedvalues	AX-184043005	2B	11149178	11940989	0.413		0.38	0.42	0.31
Shape homogeneity (UFS)	Combinedvalues	AX-184466777	2D	12871717	11774978	0.413		0.35	0.42	0.41
Firmness (FIRM)	2021	AX-184039356	6A	7277130	27533782	0.415		0.34	0.38	0.36
Glossiness (GLO)	combinedvalues	AX-184951955	4C	727143	26073661	0.436		0.38	0.35	0.37
TSS (Brix)	2020	AX-184330352	5A	22141939	18780692	0.472		0.31	0.45	0.38
Firmness (FIRM)	2021	AX-123522331	2C	22908001	3685634	0.475		0.34	0.36	0.39
Titratable acidity (TA)	2020	AX-184091372	1A	13540517	13469152	0.489		0.38	0.39	0.39
TSS (Brix)	2020	AX-184221848	6C	33293635	29374007	0.499		0.43	0.46	0.33
TSS (Brix)	2020	AX-184507945	5A	21971093	18609839	0.5		0.33	0.45	0.37
TSS (Brix)	2020	AX-184219801	5A	22266307	18905072	0.5		0.31	0.45	0.38
Glossiness (GLO)	combinedvalues	AX-184599570	3D	26901693	4775280	0.509		0.39	0.39	0.21
Skin color (COL)	Combinedvalues	AX-184090167	7C	19438028	13528856	0.535		0.35	0.35	0.34
TSS (Brix)	2020	AX-184079508	7C	26901718	19354380	0.545		0.32	0.34	0.38
TSS (Brix)	Combinedvalues	AX-184179821	5A	22384778	18999088	0.553		0.31	0.45	0.38
Skin color (COL)	2020	AX-184965421	5D	14022053	13542145	0.555		0.35	0.43	0.41
Skin color (COL)	Combinedvalues	AX-184965421	5D	14022053	13542145	0.555		0.35	0.43	0.41
Bruiseness (BRU)	combinedvalues	AX-184713611	7C	20943204	15149370	0.56		0.41	0.41	0.39
Titratable acidity (TA)	Combinedvalues	AX-184462338	6C	27458040	--	0.575		0.35	0.41	0.39
Bruiseness (BRU)	combinedvalues	AX-184940044	1A	3531536	--	0.623		0.28	0.39	0.42
Fruit weight (FW)	combinedvalues	AX-184413183	1B	19119571	15971709	0.639		0.38	0.41	0.35
Firmness (FIRM)	2021	AX-184521799	2A	17814521	5400298	0.641		0.36	0.38	0.41
B/Aratio	2021	AX-184452909	6D	1646690	32715821	0.685		0.44	0.41	0.32
TSS (Brix)	2020	AX-184477629	3B	1541393	1842542	0.757		0.37	0.43	0.38
Shape homogeneity (UFS)	Combinedvalues	AX-184477629	3B	1541393	1842542	0.757		0.37	0.43	0.38
TSS (Brix)	2020	AX-184418966	5A	24950308	21580601	0.775		0.29	0.39	0.41
B/Aratio	Combinedvalues	AX-184399755	6B	31578303	9217798	0.793		0.39	0.27	0.20
TSS (Brix)	2021	AX-184399755	6B	31578303	9217798	0.793		0.39	0.27	0.20
Skin color (COL)	Combinedvalues	AX-184965919	6A	14476176	21070121	0.799		0.29	0.36	0.38
Skin resistance (SR)	2021	AX-184230747	5B	15853776	12173853	0.804		0.35	0.39	0.41
Bruiseness (BRU)	combinedvalues	AX-184408176	5A	18814640	16414924	0.838		0.30	0.34	0.31
Fruit weight (FW)	combinedvalues	AX-184592155	2D	15565564	8801569	0.876		0.40	0.44	0.41
Shape homogeneity (UFS)	2021	AX-184458801	3A	21213134	9650845	0.88		0.30	0.36	0.43
Skin resistance (SR)	2021	AX-184127736	4A	20012930	16433479	0.916		0.36	0.34	0.34
Fruit weight (FW)	combinedvalues	AX-184241601	5B	17045086	10918733	0.925		0.32	0.30	0.34
B/Aratio	2021	AX-184052133	3B	4097709	3828069	0.937		0.34	0.39	0.35
Bruiseness (BRU)	combinedvalues	AX-184203769	1B	9439391	7276991	0.954		0.32	0.37	0.44
TSS (Brix)	2021	AX-184047575	7C	20267768	--	0.957		0.41	0.40	0.37
B/Aratio	2021	AX-184282016	3B	4095262	3825622	0.968		0.34	0.39	0.35
TSS (Brix)	2020	AX-184718481	2D	13298291	--	0.982		0.40	0.46	0.41
Shape homogeneity (UFS)	2021	AX-184554177	1A	10002081	10295193	0.994		0.30	0.34	0.35
Shape homogeneity (UFS)	2021	AX-184611387	1A	9927298	10221629	0.998		0.31	0.42	0.42
Shape homogeneity (UFS)	2021	AX-89904139	1A	9957207	--	0.998		0.30	0.34	0.35

Supplementary Table S5. Functions and/or possible roles of the 64 candidate genes underlying the trait associations.

Trait	Chromosome	Protein encoded by the Candidate Gene (CG)	CG Abbreviation	Function and/or possible role
Fruit weight	1B	cyclin-dependent kinase E-1	*CDKE*	cell division and regulation of developmental growth
	2D	small auxin upregulated RNA 14	*SAUR14*	cell differentiation and regulation of developmental growth
		small auxin upregulated RNA 1	*SAUR1*	cell differentiation and regulation of developmental growth
		small auxin upregulated RNA 20	*SAUR20*	cell differentiation and regulation of developmental growth
		small auxin upregulated RNA 51	*SAUR51*	cell differentiation and regulation of developmental growth
		small auxin upregulated RNA 49	*SAUR49*	cell differentiation and regulation of developmental growth
	5B	cullin	*CUL*	a component of SCF ubiquitin ligase complexes involved in mediating responses to auxin
		cullin	*CUL*	a component of SCF ubiquitin ligase complexes involved in mediating responses to auxin
Skin color	5C	anthocyanidin 3-O-glucosyltransferase	*FaGT2*	glycosyltransferase that transfers UDP-glucose to anthocyanidins thus generating stable 3-O-glucosides
	5D	flavonoid 3-O-glycosyltransferase	*GT*	glycosyltransferase that transfers UDP-glucose to anthocyanidins thus generating stable 3-*O*-glucosides
	6A	caffeoylshikimate esterase	*CSE*	key enzyme in the lignin biosynthetic pathway
	7B	anthocyanidin 3-O-glucosyltransferase	*FaGT1*	glycosyltransferase that transfers UDP-glucose to anthocyanidins thus generating stable 3-*O*-glucosides
	7C	TT12-like MATE transporter	*TT12*	transport of anthocyanidins to the vacuole
Firmness	3D	AGP galactosyltransferase	*GALT*	hydroxyproline-O-galactosyltransferase specific for cell wall arabinogalactan-protein biosynthesis
		* xyloglucan endotransglucosylase/hydrolase	*XTH*	catalyzes the cleavage of xyloglucans, thus functions in the loosening and rearrangement of the cell wall
		* cellulose synhase	*CES*	involved in cellulose synthesis and cell wall formation
	6A	cellulase 1	*CEL*	endo-1,4-beta-glucanase activity, involved in cell wall modifications and cell elongation
		polygalacturonase	*PG*	polygalacturonase activity that depolymerizes cell wall pectins; involved in strawberry fruit softening
		polygalacturonase	*PG*	polygalacturonase activity that depolymerizes cell wall pectins; involved in strawberry fruit softening
Titratable acid	1A	pyruvate kinase	*PK*	involved in glycolysis and in the control of citric acid during strawberry fruit ripening
	6A	V-type proton ATPase subunit G	*VMA-G*	vacuolar ATPase establishes the electrochemical gradient for proton across the tonoplast
	6C	V-type proton ATPase subunit C	*VMA-C*	vacuolar ATPase establishes the electrochemical gradient for proton across the tonoplast
TSS	1B	sucrose-phosphate synthase	*SPS*	involved in sucrose synthesis
		sucrose-phosphate synthase	*SPS*	involved in sucrose synthesis
	2D	fructose-1,6-bisphosphatase, cytosolic	*FBP*	formation of fructose-6-phosphate for sucrose biosynthesis, possible role in fructose-mediated signaling
	3B	* starch synthase	*SS*	involved in starch granule initiation and formation
	5A	fructose-1,6-bisphosphatase, cytosolic	*FBP*	formation of fructose-6-phosphate for sucrose biosynthesis, possible role in fructose-mediated signaling
	6B	fructose-bisphosphate aldolase	*FBA*	a key metabolic enzyme in glycolysis and gluconeogenesis in plants
		isocitrate dehydrogenase [NAD]	*IDH*	produces 2-oxoglutarate; key regulatory step of the TCA cycle
	6C	aconitase	*ACO*	can catalyze the conversion of citrate to isocitrate, may participate in primary metabolism including the TCA cycle
	6D	isocitrate dehydrogenase [NAD]	*IDH*	produces 2-oxoglutarate; key regulatory step of the TCA cycle
	7B	hexose carrier protein 6	*HEX*	transport of hexoses
	7C	alkaline/neutral invertase	*INV*	involved in sucrose breakdown to produce glucose and fructose
		hexose carrier protein 6	*HEX*	transport of hexoses
B/A ratio	6B	isocitrate dehydrogenase [NAD]	*IDH*	produces 2-oxoglutarate; key regulatory step of the TCA cycle
Glossiness	1C	cinnamyl-alcohol dehydrogenase	*CAD*	lignin biosynthesis
		cinnamyl-alcohol dehydrogenase	*CAD*	lignin biosynthesis
	3D	MYB-SHAQKYF	*MYS*	TF involved in wax biosynthesis; acts upstream of the DEWAX-SPL9 module with MYS2, thus regulating CER1
		trichome birefringence-like 38	*TBL*	involved in epiderm differentiation, CW modifications, pectin esterification
		* hydroxycinnamoyl-CoA shikimate/quinate hydroxycinnamoyltransferase	*HCT*	plays a critical function in the phenylpropanoid pathway in plants; is required for the formation of an intact cuticle
		* GDSL esterase/lipase	*GELP*	large gene family with several members involved in the synthesis and depolymerisation of cutin and suberin
		* GDSL esterase/lipase	*GELP*	large gene family with several members involved in the synthesis and depolymerisation of cutin and suberin
		* GDSL esterase/lipase	*GELP*	large gene family with several members involved in the synthesis and depolymerisation of cutin and suberin
	4C	non specific Lipid Transport Protein	*nsLTP*	possible transport of cuticle lipid precursors
		glycerol-3-phosphate acyltransferase	*GPAT6*	synthesis of cutin precursors
	5A	non specific Lipid Transport Protein	*nsLTP*	possible transport of cuticle lipid precursors
		glycerol-3-phosphate acyltransferase	*GPAT3*	synthesis of cutin precursors
Skin resistanc	3D	MYB-SHAQKYF 1	*MYS*	TF involved in wax biosynthesis; acts upstream of the DEWAX-SPL9 module with MYS2, thus regulating CER1
		trichome birefringence-like 38	*TBL*	involved in epiderm differentiation, CW modifications, pectin esterification
		* hydroxycinnamoyl-CoA shikimate/quinate hydroxycinnamoyltransferase	*HCT*	plays a critical function in the phenylpropanoid pathway in plants; required for the formation of an intact cuticle
		* GDSL esterase/lipase	*GELP*	large gene family with several members involved in the assembly and depolymerisation of cutin and suberin
		* GDSL esterase/lipase	*GELP*	large gene family with several members involved in the assembly and depolymerisation of cutin and suberin
		* GDSL esterase/lipase	*GELP*	large gene family with several members involved in the assembly and depolymerisation of cutin and suberin
	4A	COBRA-like	*COBL*	GPI anchored protein; key regulator of cellulose crystallinity and cell expansion
		COBRA-like	*COBL*	GPI anchored protein; key regulator of cellulose crystallinity and cell expansion
		beta-D-xylosidase	*BXL*	required for arabinan modification in CW pectins
		trichome birefringence-like 43	*TBL*	involved in secondary wall cellulose deposition, presumably through the esterification state of pectic polymers
	5B	pectin methylesterase inhibitor	*PMEI*	inhibit pectin methylesterase activity thus leading to a higher degree of methylesterification of pectin
	7A	polygacturonase	*PG*	polygalacturonase activity that depolymerizes cell wall pectins
		expansin B2	*EXP*	mediates cell wall loosening and therefore modulates cell wall strength and structural integrity
		expansin B2	*EXP*	mediates cell wall loosening and therefore modulates cell wall strength and structural integrity
		decrease was biosynthesis 2	*DEWAX*	DEWAX2 Transcription Factor Negatively Regulates Cuticular Wax Biosynthesis in Arabidopsis Leaves
		decrease was biosynthesis 2	*DEWAX*	DEWAX2 Transcription Factor Negatively Regulates Cuticular Wax Biosynthesis in Arabidopsis Leaves
	7A	wax ester synthase	*WSD*	involved in wax metabolism
Bruiseness	1A	epidermal patterning factor	*EPFL*	plant specific secretory peptides with roles in the control of patterning in the plant epidermis
	1B	GDSL lipase	*GELP*	large gene family with several members involved in the assembly and depolymerisation of cutin and suberin
	5A	pectate lyase	*PL*	cleaves pectin homogalacturonan - involved in strawberry fruit softening
	6B	fasciclin-like arabinogalactan protein	*FLA*	involved in cell wall formation and architecture
		fasciclin-like arabinogalactan protein	*FLA*	involved in cell wall formation and architecture
		xyloglucan endotransglucosylase/hydrolase	*XTH*	catalyzes the cleavage of xyloglucans, thus functions in the loosening and rearrangement of the cell wall
	7C	3-ketoacyl-CoA synthase 1-like	*KCS*	involved in cuticular wax synthesis; possibly involved in fruit water-loss after harvest
